# Tirzepatide in dermatology: cutaneous adverse events, emerging therapeutic roles, and cosmetic implications – A comprehensive review^☆^

**DOI:** 10.1016/j.abd.2025.501255

**Published:** 2025-12-24

**Authors:** Heba Saed El-Amawy

**Affiliations:** Department of Dermatology and Venereology, Faculty of Medicine, Tanta University, Tanta, Gharbia, Egypt

**Keywords:** Glucagon-like peptide-1 receptor agonists, Skin, Tirzepatide, Tirzepatide /adverse effects, Tirzepatide/therapeutic use

## Abstract

Tirzepatide, a dual Glucose-dependent Insulinotropic Polypeptide (GIP) and Glucagon-Like Peptide-1 (GLP-1) receptor agonist. Tirzepatide was first approved by the FDA for type 2 diabetes in May 2022 and subsequently for obesity in November 2023, and has demonstrated significant efficacy in glycemic control and weight reduction. Beyond its metabolic benefits, recent evidence highlights its relevance in dermatology. This review explores the dermatologic implications of tirzepatide, including its cutaneous adverse effects, therapeutic potential in inflammatory skin diseases, and cosmetic benefits. Cutaneous side effects such as hypersensitivity reactions, injection-site reactions, and rare severe dermatologic events have been documented. Across the SURPASS clinical trials, injection-site reactions occurred slightly more frequently, comparable to other GLP-1 receptor agonists as semaglutide. Meanwhile, tirzepatide's immunomodulatory properties suggest potential therapeutic roles in conditions like psoriasis and hidradenitis suppurativa; however, current evidence is limited to case reports and small studies. Additionally, its profound effects on fat distribution raise interest in its cosmetic implications. Tirzepatide's induced rapid weight loss may lead to aesthetic changes, including facial volume loss, which warrants cautious interpretation. This narrative review summarizes current data from clinical trials, case reports, and pharmacovigilance sources, based on a literature search of PubMed, Scopus, and Google Scholar up to May 2025, focusing on skin-related adverse events, therapeutic effects, and cosmetic outcomes of tirzepatide.

## Introduction

Tirzepatide is a new medication designed and approved by the US Food and Drug Administration (FDA) in May 2022 (Mounjaro) to treat type 2 diabetes and approved for obesity treatment in November 2023 (Zepbound).^1^ It works by simulating the action of two natural gut hormones, GIP (Glucose-dependent Insulinotropic Polypeptide) and GLP-1 (Glucagon-Like Peptide-1), which are released after eating and are responsible for appetite, calorie intake control, and regulation of food intake and therefore they contribute to the regulation of blood sugar levels. Because it activates both receptors, tirzepatide is known as a dual incretin receptor agonist.^2^

## Methodology

This narrative review was conducted through a comprehensive search of the literature from inception to May 2025 across PubMed, Scopus, and Google Scholar. Search terms included combinations of keywords and Boolean operators, such as (“tirzepatide” OR “Mounjaro” OR “Zepbound”) AND (“GLP-1 receptor agonist” OR “dual incretin”) AND (“dermatology” OR “skin” OR “cutaneous” OR “adverse effects” OR “hypersensitivity” OR “injection-site reactions” OR “cosmetic”). Medical Subject Headings (MeSH) were applied in PubMed where available (e.g., “Skin Diseases,” “Drug-Related Side Effects and Adverse Reactions”). A representative search string in PubMed was: ("tirzepatide"[Mesh] OR "tirzepatide"[tiab]) AND ("Skin Diseases"[Mesh] OR "cutaneous"[tiab] OR "dermatology"“tirzepatide”[Mesh] OR “tirzepatide”[tiab]) AND (“Skin Diseases”[Mesh] OR “cutaneous”[tiab] OR “dermatology”[tiab]. In Google Scholar, the first 200 results were screened, and only peer-reviewed articles were included. Eligible studies were English-language original research, reviews, clinical trials, and case reports addressing dermatologic adverse effects, therapeutic implications, or cosmetic outcomes. Duplicates were removed manually, and references were hand-searched for additional studies. Screening and extraction were performed by the author, and findings were synthesized narratively due to the limited scope of available evidence.

### Chemical structure of tirzepatide

The molecule is a synthetic polypeptide made up of 39 amino acids, based mainly on the GIP sequence, with some modifications that allow it to also activate the GLP-1 receptor. To prolong its half-life and allow once-weekly dosing, a C20 fatty diacid side chain is conjugated to the peptide, promoting albumin binding.^3^

The difference between tirzepatide and other medications is its ability to act on two different receptors at the same time. GLP-1 receptor agonists, such as exenatide, liraglutide, dulaglutide, and semaglutide, which only stimulate the GLP-1 pathway, help lower blood sugar and reduce appetite, but their effect is limited to one pathway. In contrast, tirzepatide’s dual action results in greater improvements in both blood sugar control and weight loss, as seen in recent clinical trials. It also appears to be better tolerated in some patients because the GIP part of the molecule may help reduce common side effects like nausea and vomiting. Overall gastrointestinal tolerability seems broadly comparable to GLP-1 receptor agonists; however, the suggestion that GIP receptor activation may attenuate nausea remains speculative and requires further validation.^3^, ^4^

### Mechanism of action of tirzepatide

Tirzepatide is a novel dual receptor agonist that targets both the glucose-dependent insulinotropic polypeptide and glucagon-like peptide-1 receptors. Studies reveal a greater degree of engagement of tirzepatide for the GIP receptor than the GLP-1 receptor, so the molecule demonstrates higher affinity for the GIP receptor, with partial agonism at the GLP-1 receptor.^5^ Upon subcutaneous administration, tirzepatide binds to and activates both receptors, leading to enhanced insulin release from the pancreas, suppression of glucagon release, slowing of gastric emptying, and promotion of satiety by central mechanisms, thereby improving postprandial and overall glycemic control. Furthermore, GIP receptor activation provides additional metabolic benefits, such as improved adipocyte insulin sensitivity, and may also counteract the gastrointestinal side effects often associated with GLP-1, thus enhancing tolerability and adherence to therapy; however, these hypotheses require further validation The synergistic action of these pathways results in significant reductions in HbA1c and substantial weight loss, compared to those observed with GLP-1 receptor agonists alone. Additionally, tirzepatide has also been shown to increase adiponectin levels, a protein hormone that regulates glucose and fatty acid oxidation. This may contribute to favorable effects on insulin sensitivity and lipid metabolism, but current evidence remains limited. In the SURPASS clinical program, tirzepatide achieved superior reductions in HbA1c compared with GLP-1 receptor agonists, while the SURMOUNT trials demonstrated substantial weight loss in individuals with obesity.^4^, ^6^ Although these results highlight the therapeutic potential of dual agonism, claims of cardiovascular risk reduction should remain cautious until dedicated Cardiovascular Outcome Trials (CVOTs) are fully published.^1^, ^4^, ^7^, ^8^

### Pharmacokinetics and efficacy of tirzepatide

Upon injection, tirzepatide is metabolized into single amino acids in different tissues, including the liver, where the polypeptide structure undergoes proteolytic cleavage, and the fatty acid side chain undergoes oxidation and amide hydrolysis. This fatty acid chain helps tirzepatide bind to albumin, slowing its degradation and clearance. Tirzepatide takes about 8‒72 hours to reach a peak serum level. It has a half-life of 5-days, which allows for the weekly subcutaneous injection. The drug metabolites are cleared in urine and feces.^9^ The earlier reports categorized tirzepatide as a “second-line” therapy following GLP-1 receptor agonists.^10^ However, current ADA (American Diabetes Association) 2024 and SBD (Sociedade Brasileira de Diabetes) 2024 guidelines support its early use in selected patients based on clinical characteristics and treatment goals rather than as an unconditional first-line agent.^11^, ^12^

When comparing to placebo in SURPASS-1, there was a -2.11% reduction in HbA1c levels at 5 mg per week dosing with a 5.4 kg weight reduction compared to -0.86% with a placebo, which increased to a -2.34% reduction in HbA1c and 10.5 kg weight reduction when reaching the highest dose of 15 mg per week of tirzepatide.^4^ In SURPASS-2 (head-to-head vs. semaglutide), tirzepatide produced superior reductions in both HbA1c and body weight.^13^ In SURPASS-5, tirzepatide reduced HbA1c by up to -2.34% and body weight by -10.5 kg over 40-weeks in a dose-dependent manner. These effects are comparable to semaglutide for glycemic and weight management.^14^ When administered to nondiabetic patients, in SURMOUNT-1 (obesity without diabetes), in a dose of 5 to 15 mg once weekly, tirzepatide produced remarkable reductions in weight, ranging from 16.5% to 22.4% over a period of 72-weeks.^7^ Owing to the weight loss action, it is likely to have an indirect effect in the treatment of nonalcoholic fatty liver disease; however, this indication remains investigational.^15^

### Dosage and adverse effects of tirzepatide

Tirzepatide is administered once weekly by subcutaneous injection, with the dose gradually increased based on HbA1c, weight response, and tolerability. The initial 2.5 mg dose is for tolerability rather than glycemic efficacy, so treatment typically starts at 2.5 mg once weekly, then after 4-weeks, the dose is increased to 5 mg, with further increases of 2.5 mg at ≥ 4-week intervals if needed. The maximum dose is 15 mg weekly. Evidence of dose modification in end-stage renal disease and advanced liver disease is limited, and use during pregnancy or lactation should be avoided due to insufficient safety data.^16^

Tirzepatide is generally well-tolerated, with most side effects being dose dependent. The most common side effects are gastrointestinal, including nausea, diarrhea, vomiting, constipation, and decreased appetite, particularly during the initial weeks of treatment, which improve over time. Hypoglycemia due to tirzepatide is rare unless tirzepatide is combined with insulin or sulfonylureas. Pancreatitis has been reported, but causality is unproven. Gallbladder disease (e.g., cholelithiasis) and acute kidney injury have been described as associations, often related to dehydration from GI losses. Overall, the safety profile of tirzepatide closely resembles that of GLP-1 receptor agonists, with careful monitoring recommended, especially during dose titration.^2^, ^17^

Tirzepatide should not be used with other GLP-1 agonists and must be used cautiously with insulin to avoid hypoglycemia. It can reduce the effectiveness of oral contraceptives and affect the absorption of other oral drugs due to delayed gastric emptying. Contraindications include a personal or family history of medullary thyroid carcinoma, MEN-2 (Multiple endocrine neoplasia type 2), and known hypersensitivity to the drug. Routine monitoring should include HbA1c, weight, appearance of signs of adverse effects such as GI upset and pancreatitis, and new thyroid nodules on therapy.^13^, ^18^

Due to its widespread global use and subcutaneous route of administration, there is a strong need to better understand the cutaneous adverse effects associated with tirzepatide. While tirzepatide’s metabolic benefits have been well documented, adverse effects are also reported, with cutaneous reactions needing to be highlighted. These skin-related side effects range from mild injection site reactions to rare but potentially serious hypersensitivity responses. This review aims to provide a detailed overview of tirzepatide-induced cutaneous side effects, suggested underlying mechanisms, clinical implications, and management strategies. The reported skin-related side effects to tirzepatide are summarized in Table 1.

**Table 1:** Reported dermatologic adverse events associated with tirzepatide.

Year	Study Type	Dermatologic Adverse Event	Number of Patients Affected	Dosage	Time of Onset	Management	Mechanism Explanation
2021^13^	SURPASS-2 (Phase 3 RCT)	Injection-site reaction and hypersensitivity reactions	Injection site reactions: 1.9% (5 mg), 2.8% (10 mg), 4.5% (15 mg) Hypersensitivity reactions: 1.7%‒2.8%	5 mg, 10 mg, 15 mg weekly	Not specified	Symptomatic management, often self-limited	Possibly related to local immune response to subcutaneous injection and/or presence of anti-drug antibodies
2021^20^	SURPASS-3 (Phase 3 RCT)	Injection-site reaction and hypersensitivity reactions	ISRs: <1%–2% Hypersensitivity reactions: ∼3% for all doses	5 mg, 10 mg, 15 mg weekly	Not specified	Often mild and self-resolving	Local immune response; similar rate to insulin degludec
2023^24^	Case Report	Injection site rash	1	2.5 mg weekly	10-days post-injection	Discontinuation of tirzepatide induced resolution within 1 month	Not specified
2024^25^	Cross-Sectional Analysis	Rash, pruritus, alopecia, urticaria, hyperhidrosis	Overall skin related side effects of tirzepatide (14.22%): Rash (17.7%), Pruritus (12.9%), Hyperhidrosis (6%), urticaria (14.9%) and Alopecia (22.7%)	Not specified	Not specified	Not specified	Not specified
2023^27^	Case Report	Biphasic anaphylactic reaction (diffuse urticarial rash and angioedema as well as respiratory and cardiovascular collapse), Recurrence of symptoms of anaphylaxis after an initial resolution	1	5 mg weekly	20 minutes post-injection	Epinephrine, methylprednisolone, diphenhydramine; observation in ER (It is suggested that patients with anaphylaxis to tirzepatide might benefit from desensitization therapy)	IgE-independent anaphylaxis (there was no prior exposure to tirzepatide)
2024^29^	Case Report	Immediate-type allergic reaction to tirzepatide: A sudden onset of severe disseminated pruritus and a generalized urticarial rash on arms, hands, back, and entire body, excluding face and neck	1	Not specified (but occurred after the first injection of tirzepatide)	10–15 minutes post-injection	Antihistamines, symptoms resolved upon discontinuation	Possible IgE-mediated hypersensitivity
2025^32^	Retrospective Analysis	The most common: Eczematous reactions, pruritus, drug eruptions, hyperhidrosis, alopecia	Overall, 690 reactions (5.96%) of the total reported cutaneous adverse events: Eczematous reactions (183 cases), alopecia (155 cases), drug eruption (151 cases), pruritus (130 cases), hyperhidrosis (39 cases), skin discoloration (8 cases), life threatening reactions (7 cases), psoriasis (4 cases), acne (5 cases), nail changes (3 cases), photosensitivity (4 cases)	Not specified	Not specified	Not specified	Not specified

### Injection site reactions

Injection Site Reactions (ISRs) are the most frequently notable tirzepatide-associated skin-related side effects.^14^, ^19^ These ISRs typically present as localized redness and erythema around the injection area, mild swelling or firmness and induration, pruritus, and discomfort or pain during and after the injection at the injection site. Across the SURPASS clinical trials, injection site reactions with tirzepatide were consistently reported in approximately 2%–6% of patients, compared to ≤ 1% with placebo or active comparators (semaglutide, dulaglutide, insulin degludec, or glargine).^4^, ^13^, ^14^, ^19^, ^20^, ^21^ Also, according to the FDA prescribing information, injection-site reactions were reported more frequently in patients who developed anti-tirzepatide antibodies (4.6%) compared to patients without antibodies (0.7%).^22^

Although incidence appeared slightly higher in patients who developed anti-tirzepatide antibodies, this represents a correlation rather than established causality. Mullin et al., reported that hypersensitivity and injection site reactions were more frequent in Treatment Emergent (TE) Anti-Drug Antibodies (ADA)+ than in TE ADA- patients, no consistent temporal association was detected between the time of the event reporting and ADA status, and most events resolved irrespective of ADA status.^19^

In the SURMOUNT obesity trials, ISRs rates were slightly higher, occurring in about 6%–8% of those patients who used tirzepatide for weight management, compared to 2% in the placebo group. Also, the reactions were usually mild and resolved on their own within a few days.^6^, ^7^, ^23^ The risk of ISRs is consistent with other injectable incretin therapies. ISRs likely result from a localized immune or inflammatory response to the peptide, excipients, or injection trauma. Repeated injections in the same area can increase the risk of irritation.^19^, ^24^, ^25^ The important differential diagnosis of ISRs includes cellulitis, which could be excluded by the self-limited course of the symptoms and the absence of fever and systemic symptoms.^24^ Patient education on site rotation and aseptic technique is essential to minimize recurrence and complications.

### Hypersensitivity reactions

In addition to the local injection site reactions, tirzepatide may cause generalized skin rashes such as maculopapular eruptions, urticarial eruptions, and, in rare cases, more serious conditions like angioedema; despite being rare, but have been reported. Across the SURPASS studies, hypersensitivity reactions occurred in approximately 1%–2% of tirzepatide-treated patients, slightly higher than the 1% reported in placebo groups, and were generally mild to moderate in severity.^4^, ^13^, ^14^ In immunogenicity analysis study by Mullin et al., that analyzed seven Phase 3 SURPASS studies, the rate was somewhat higher (∼3–4%), with greater incidence in antibody-positive patients (4.1% vs. 3.0% in antibody-negative), though this difference was not clinically meaningful and data on treatment-emergent antidrug antibodies and hypersensitivity remain inconsistent across studies, with no clear causal link established.^19^ Furthermore, patients who developed treatment-emergent antidrug antibodies did not experience severe hypersensitivity or injection-site reactions, though comprehensive data were lacking.^19^, ^26^ Importantly, hypersensitivity is recognized as a class effect of GLP-1 receptor agonists, and vigilance is required.^25^ Patients should be counseled on the signs of systemic reactions such as angioedema or anaphylaxis and instructed to seek urgent medical attention if these occur.^19^

### Severe allergic reactions

Although extremely rare, severe allergic reactions, including anaphylaxis and serum sickness-like cutaneous and systemic symptoms, have been linked to tirzepatide. These require immediate medical attention. The FDA has issued warning labels for Mounjaro and Zepbound, highlighting these risks and advising patients to be vigilant for symptoms like facial swelling, breathing difficulty, or widespread rash.^22^, ^27^, ^28^

The exact mechanisms of tirzepatide-induced cutaneous side effects are not fully elucidated, but several hypotheses exist. Firstly, the immunogenicity of the tirzepatide synthetic peptide, as tirzepatide may be recognized as foreign by the immune system, triggering local or systemic immune responses.^25^ Formation of anti-drug antibodies was more evident in patients who developed hypersensitivity and injection site reactions as described by Mullins et al.^19^ Secondly, the injection itself can include minor tissue trauma, activating local inflammatory reaction and releasing mediators and cytokines. Thirdly, patients with pre-existing allergies or sensitivities to GLP-1 analogs or tirzepatide itself might be predisposed to IgE-dependent skin reactions when exposed again to tirzepatide due to structural similarities as a form of cross-reactivity.^27^, ^29^ Lastly, the preservatives or other inactive ingredients might stimulate allergic contact dermatitis or irritant reactions. However, mechanistic hypotheses such as immunogenicity, antidrug antibodies, or T–cell–mediated responses remain speculative. Antibody development is usually non-neutralizing and shows no consistent effect on pharmacokinetics, efficacy, or safety.^19^ Reported hypersensitivity and ISR differences in antibody-positive versus antibody-negative patients are small and need further correlations.

Clinically, to differentiate, injection site reactions usually appear within hours, are usually localized and limited to areas like the abdomen, thigh, or upper arm, and they are self-limiting, resolving within 1‒3 days, while tirzepatide-induced hypersensitivity reactions can occur days to weeks after starting therapy and may include generalized itching, urticarial rash, or angioedema. Severe cases may involve fever, malaise or breathing difficulty. Diagnosis is mainly clinical and based on the timing of symptom onset after drug initiation.^24^, ^29^ To reduce the risk of skin-related side effects of tirzepatide, patients should be aware of the common skin reactions. It is better to advise the patients to rotate injection sites and use the correct injection technique. Mild reactions can be treated with cold compresses, antihistamines, or topical corticosteroids. Severe reactions may require stopping the drug and starting corticosteroids. Systemic corticosteroids should be reserved for selected severe or prolonged reactions, while intramuscular epinephrine remains the first-line emergency treatment for anaphylaxis. Regular monitoring and reporting of skin reactions help improve understanding and safety of tirzepatide use.^24^, ^27^, ^30^

### Skin side effect profile: Tirzepatide versus GLP-1 analogues

Regarding the skin-related side effects of semaglutide, a recent review analyzed 22 studies involving 255 patients. The common dermatologic adverse events included injection site reactions (3.5%), which were more frequent with placebo (6.7%), and higher rates of altered skin sensations such as paresthesia, dysesthesia, and burning sensation, especially with a high dose of oral semaglutide (50 mg weekly). Alopecia has been reported (up to 6.9% in one study of oral semaglutide), compared to 0.3% on placebo, though causality remains unproven, representing an emerging signal requiring further study. Two cases of keratinocyte carcinoma, squamous and basal cell carcinoma, were reported among 258 patients at risk receiving semaglutide, while no cases were observed in the placebo or the comparison groups. Rare but severe side effects included angioedema, bullous pemphigoid, leukocytoclastic vasculitis, and eosinophilic fasciitis, which led to discontinuation of the drug. The study highlights the need for awareness of these potential dermatologic reactions and calls for further research to understand their mechanisms and risk factors.^31^ Therefore, similar injection site and hypersensitivity reactions are observed with GLP-1 receptor agonists like liraglutide, semaglutide, and dulaglutide, and tirzepatide does not appear to increase the incidence or severity of cutaneous side effects compared to these agents.^4^, ^30^

In the SURPASS-2 and SURPASS-3 clinical trials, both hypersensitivity and injection-site reactions were observed with tirzepatide treatment, with some differences compared to semaglutide and insulin degludec. Injection-site reactions were more frequent with tirzepatide, particularly in the SURPASS-2 trial, where rates increased with dose (1.9% at 5 mg, 2.8% at 10 mg, and 4.5% at 15 mg) compared to only 0.2% with semaglutide.^13^ In contrast, in the SURPASS-3 trial, the incidence of injection-site reactions with tirzepatide was lower (<1%–2%) and comparable to insulin degludec (2%). Hypersensitivity reactions with tirzepatide occurred at rates of 1.7% to 2.8% in SURPASS-2, similar to semaglutide (2.3%), and consistently at 3% across all tirzepatide doses in SURPASS-3, higher than insulin degludec (1%).^20^ These findings suggest that while tirzepatide may cause more frequent local injection-site reactions than semaglutide, its hypersensitivity risk appears mild and generally comparable to other injectable therapies.^26^

Furthermore, a cross-sectional analysis of adverse dermatologic events reported to the FDA after the use of GLP-1 agonists, including semaglutide and tirzepatide, found that dermatologic adverse events were reported in a percentage of 6.08% (4,896) of a total of 80,482 adverse events. Liraglutide accounted for the highest proportion of these skin-related events (45.85%), followed by semaglutide (40.18%) and tirzepatide (14.22%). Notably, there was a 186% rise in dermatologic reports from 2022 to 2023, aligning with the broader commercial use of these drugs. The most common dermatologic reactions were rash (21.79%), pruritus (17.95%), alopecia (13.97%), urticaria (11.09%), and hyperhidrosis (10.76%). While all three GLP-1 receptor agonists shared these top five reactions, their frequency varied. Tirzepatide is associated with a lower frequency of rash, pruritus, and hyperhidrosis, but a higher frequency of alopecia and urticaria compared to liraglutide and semaglutide. Reports of lipodystrophy and lipoatrophy were rare, with only five combined cases.^25^ These findings highlight unique dermatologic safety considerations for tirzepatide, which may be relevant when counseling patients on treatment options. It should be noted that the FDA Adverse Event Reporting System (FAERS) data are subject to reporting bias and cannot establish incidence or causality.

The findings of Mullins et al., in their comprehensive analysis of patients treated with tirzepatide in seven Phase 3 studies, revealed that 51.1% of patients developed ADA during the treatment period. Notably, hypersensitivity reactions occurred in 4.1% of ADA-positive patients, compared to 3.0% in ADA-negative patients. Similarly, injection site reactions were reported in 4.6% of ADA-positive patients, versus 0.7% in those without ADA, suggesting that immunogenicity may contribute to these adverse events. These reactions were predominantly mild to moderate in severity and resolved independently of ADA status or titer levels. Importantly, the development of ADA did not impact the pharmacokinetics or efficacy of tirzepatide, indicating that immunogenicity did not adversely affect the drug's performance.^19^

In the same context, a retrospective review of cutaneous adverse events associated with semaglutide, dulaglutide, tirzepatide, lixisenatide, liraglutide, and exenatide found that the five most common cutaneous reactions associated with GLP-1 agonists were eczematous, pruritus, drug eruptions, hyperhidrosis, and alopecia. Life-threatening cutaneous adverse events accounted for 2.17% of all cutaneous reactions, with no statistically significant differences observed between drug types. Tirzepatide is associated with a lower overall rate of reported skin-related adverse reactions compared to other GLP-1 agonists, particularly exenatide, dulaglutide, and liraglutide. Tirzepatide accounted for 690 reactions, which is 5.96% of the total reported cutaneous adverse events, much lower than exenatide (42.4%), dulaglutide (20.1%), and liraglutide (16.9%). Regarding specific skin effects of tirzepatide, the most reported adverse reaction was eczematous reactions (183 cases), followed by alopecia (155 cases), drug eruption (151 cases), pruritus (130 cases), hyperhidrosis (39 cases), skin discoloration (8 cases), and life-threatening reactions (7 cases). Compared to other agents, tirzepatide had significantly fewer reports of pruritus, hyperhidrosis, and drug eruptions. Reports of rare cutaneous events such as psoriasis, acne, nail changes, and photosensitivity were anecdotal and extremely infrequent. These retrospective findings are suggestive of a relatively favorable cutaneous safety profile for tirzepatide, though not definitive.^32^

### Aesthetic changes associated with tirzepatide use

Rapid or massive weight loss, induced by agents like semaglutide and tirzepatide, or after bariatric surgery, is associated with a significant reduction in subcutaneous fat tissue volume, predisposing to tissue laxity. This is caused by the loss of mechanical support provided by fat, which can lead to significant changes in skin structure, including decreased collagen synthesis and altered collagen fiber composition, and contribute to reduced skin firmness and increased laxity. Studies show a decrease in thick collagen fibers and an increase in thin collagen fibers, with a rise in elastic fiber density to maintain skin elasticity. The loss of underlying fat and impaired collagen cross-linking weakens the extracellular matrix, reducing mechanical skin stability and causing skin sagging and folds. Additionally, skin is directly affected by the diminished absorption of various nutrients and vitamins, such as vitamin A and D, that are crucial for collagen synthesis, and reductions in key enzymes involved in collagen production and persistent inflammation after weight loss further degrade skin integrity and elasticity, especially in areas like the abdomen, arms, and thighs.^33^, ^34^, ^35^

Facial lipoatrophy associated with GLP–1–induced weight loss (popularly termed ‘Ozempic face’) is described as the characteristic facial changes seen in some individuals using Ozempic (semaglutide). These changes typically include facial volume and fat loss, skin sagging, hollowing of the cheeks and temples, more prominent nasolabial folds and jawline, and an accelerated aging appearance. These aesthetic changes are secondary to rapid weight loss and not a direct pharmacologic toxicity of the drug, as patients on semaglutide can lose around 10.9% of their body weight in six months.^36^, ^37^, ^38^ It is not a direct pharmacologic side effect of semaglutide, but it is a severely annoying dermatological cosmetic compromise.^38^ Semaglutide-induced fat loss can significantly affect collagen synthesis, skin elasticity, alter dermal structure, and induce changes in general dermal health and skin appearance. Fat loss also alters adipokine levels and inflammatory responses, further impacting skin elasticity. However, mechanistic explanations, such as altered adipokines, inflammatory responses, and impaired collagen metabolism, remain speculative. Some studies observed regional fat loss in facial areas, resulting in noticeable aesthetic changes, but more studies are needed to fully understand semaglutide’s direct effects on skin.^39^, ^40^

Although the pharmacological treatments of obesity offer a balanced alternative to surgery and promote a gradual fat loss, which helps maintain skin integrity, reduced food intake due to drugs like tirzepatide and semaglutide may still lead to nutrient deficiencies, such as vitamin D, B12, and protein, affecting skin health, making dietary support essential during treatment. Tirzepatide has been shown to produce greater weight loss compared to semaglutide. In clinical trials, patients treated with tirzepatide are more likely to achieve 10% or greater, and 15% or greater weight loss and experience larger reductions in body weight at 3-, 6-, and 12-months.^4^, ^41^ Therefore, tirzepatide is also suspected to cause facial fat loss and changes in skin elasticity and collagen synthesis due to rapid weight loss. Patients should be made aware of these potential side effects to better manage expectations and seek appropriate care if needed.

These aesthetic changes not only affect physical appearance but also have a profound psychosocial impact, including reduced self-esteem, dissatisfaction with body image, and emotional distress. Despite the focus on metabolic and weight-related outcomes, skin laxity remains an under-recognized consequence that can undermine treatment satisfaction and long-term adherence. To address this issue, an integrated therapeutic approach is essential, including the use of collagen biostimulators such as Poly-L-Lactic Acid (PLLA) and Calcium Hydroxyapatite (CaHA) to restore skin firmness by stimulating collagen production, complemented by energy-based devices like High-Intensity Focused Ultrasound (HIFU), fractional radiofrequency, and CO_2_ laser. While no large-scale studies have yet combined tirzepatide therapy with anti-laxity treatments, clinical evidence supports their synergistic use in patients undergoing substantial weight loss. This multidisciplinary strategy ensures not only metabolic improvement but also preservation of aesthetic and psychological well-being.^40^, ^42^ These management strategies are extrapolated from bariatric surgery literature and represent expert opinion, as no controlled studies have evaluated them in tirzepatide-treated patients.

### Dermatological therapeutic benefits of tirzepatide

Tirzepatide, by significantly improving glycemic control in patients with type 2 diabetes, may lead to a reduction in several diabetes-related cutaneous manifestations. Chronic hyperglycemia is known to contribute to a variety of skin conditions, such as diabetic dermopathy, necrobiosis lipoidica, acanthosis nigricans, and increased susceptibility to infections due to impaired immune response and poor circulation. With tirzepatide’s dual GIP and GLP-1 receptor agonism, patients can achieve substantial improvements in HbA1c and insulin sensitivity, which can enhance skin barrier function, promote wound healing, and reduce the occurrence of skin infections. Clinical observations have noted improvements in conditions such as candidiasis and bacterial folliculitis with better glycemic control. Furthermore, improved metabolic profiles may also alleviate pruritus and reduce skin dryness, both commonly reported in diabetic patients. GLP-1 agonists are suggested to exert potential immunomodulatory effects, such as reducing proinflammatory cytokines and altering immune cell populations; however, these remain speculative rather than established. Therefore, tirzepatide’s metabolic benefits can extend beyond glucose regulation and may positively impact skin health in individuals with diabetes.^7^, ^43^

Anecdotal evidence from case reports has suggested possible dermatological benefits. A recent case report described significant hair regrowth with improvement in hair density within 6-months of tirzepatide at a dose of 2.5 mg weekly for the first 3-months followed by an increase of 5 mg weekly, in addition to improvement in insulin resistance and weight loss in a male patient with androgenic alopecia, suggesting that improved insulin sensitivity through tirzepatide might promote hair growth, control dihydrotestosterone levels, and release miniaturization of the dermal papilla of hair follicles.^44^ Similarly, tirzepatide induced remarkable improvement in a male patient with a 30-year history of recalcitrant Folliculitis Decalvans (FD) with symptom relief and hair regrowth, following the initiation of tirzepatide for weight management. The case highlighted that tirzepatide could help to restore immune tolerance to follicular microbiota, and also pointed to the potential anti-inflammatory and immunomodulatory properties of tirzepatide in managing FD, a chronic and refractory dermatologic disorder. This single case highlights a potential anti-inflammatory and immunomodulatory effect, though causality remains unproven, and controlled studies are needed.^45^ Additionally, a recent case report documented significant improvements in Hidradenitis Suppurativa (HS) severity and dermatology quality of life scores following combined treatment with tirzepatide, initiated at 2.5 mg/0.5 mL weekly and increased to 7.5 mg/0.5 mL weekly, and infliximab.^46^ Given the anecdotal nature of these reports, their findings should be interpreted with caution. HS is a chronic skin disorder where obesity is prevalent in up to 75% of patients, aiding in the systemic inflammatory state and a higher body mass index is positively correlated with disease severity.^47^

GLP-1 receptor agonists are suspected to have a potential therapeutic role in inflammatory skin diseases such as psoriasis and HS. In psoriasis, these agents have been linked to significant reductions in Psoriasis Area and Severity Index (PASI) scores, likely through the downregulation of pro-inflammatory cytokines like TNF-α and modulation of immune pathways, including the enhancement of peripheral regulatory T-cell function. In HS, GLP-1 agonists have been associated with decreased systemic inflammation and improved patient quality of life. These findings support further exploration of GLP-1 agonists, including tirzepatide, as adjunctive treatments in dermatologic conditions characterized by chronic inflammation.^31^, ^48^, ^49^ However, these observations remain preliminary, and controlled studies are essential before definitive conclusions can be drawn.

Lipodystrophy syndromes are characterized by visible lipoatrophy and abnormal fat redistribution, which manifest dermatologically as acanthosis nigricans, eruptive xanthomas, or impaired wound healing. These dermatologic manifestations often precede or accompany profound metabolic complications such as severe insulin resistance, hypertriglyceridemia, and hepatic steatosis.^50^ Importantly, while metreleptin remains a cornerstone for generalized forms, its efficacy in partial lipodystrophy is limited, underscoring the relevance of alternative therapies. Dermatologists frequently encounter lipodystrophy within the context of connective tissue disease-associated forms (e.g., juvenile dermatomyositis–related acquired generalized lipodystrophy) and inherited syndromes with cutaneous stigmata.^51^ Tirzepatide improves metabolic control by enhancing insulin sensitivity, stimulating glucose-dependent insulin secretion, suppressing glucagon, delaying gastric emptying, and reducing ectopic lipid accumulation. Recently, in an observational cohort of 17 patients with partial and generalized lipodystrophy, treatment with tirzepatide over a median of 8.7-months led to significant reductions in BMI (-1.7 kg/m^2^), HbA1c (-1.1%), triglycerides (-65 mg/dL), and insulin requirements (-109 units/day), with only mild gastrointestinal side effects. These metabolic improvements are directly relevant to dermatology, as better glycemic and lipid regulation may alleviate secondary cutaneous sequelae of lipodystrophy, underscoring tirzepatide’s promise as an adjunctive therapy within interdisciplinary management of lipodystrophy.^52^

Overall, dermatological benefits of tirzepatide, which are summarized in Table 2, remain an emerging area of investigation. Interdisciplinary collaboration between dermatology and endocrinology is encouraged to better define its potential role in skin disease management.

**Table 2:** Dermatological therapeutic benefits of tirzepatide.

Dermatologic Condition	Proposed Mechanism	Clinical Evidence	Remarks
Diabetes-related cutaneous manifestations (dermopathy, necrobiosis lipoidica, acanthosis nigricans, infections, pruritus, xerosis)	Improved glycemic control, enhanced insulin sensitivity, better circulation, reduced proinflammatory cytokines, possible immune modulation	Observational data & indirect evidence from improved HbA1c, triglycerides, weight^7^, ^43^	Improvement likely secondary to metabolic effects; immunomodulation still speculative
Hair disorders (androgenetic alopecia)	Improved insulin sensitivity, weight reduction, potential impact on dihydrotestosterone and release dermal papilla miniaturization	Case report: significant hair regrowth with improvement in hair density within 6-months of tirzepatide at a dose of 2.5 mg weekly for the first 3-months followed by an increase of 5 mg weekly, in addition to improvement in insulin resistance and weight loss in a male patient with androgenic alopecia^44^	Anecdotal; causality not proven
Folliculitis decalvans	Possible restoration of immune tolerance to follicular microbiota, anti-inflammatory and immunomodulatory properties	Case report: significant improvement and hair regrowth after tirzepatide initiation with symptoms relief^45^	Single case; controlled studies lacking
Hidradenitis suppurativa	Weight reduction, decreased systemic inflammation, improved metabolic profile; potential synergy with biologics (e.g., infliximab)	Case report: Marked improvements in HS severity and dermatology quality life scores following combined treatment with tirzepatide, initiated at 2.5 mg/0.5 mL weekly and increased to 7.5 mg/0.5 mL weekly, and infliximab^46^	Promising, but based on anecdotal data
Psoriasis	Downregulation of TNF-α, modulation of immune pathways, increased Treg activity	Evidence with GLP-1 agonists; limited but suggestive relevance to tirzepatide^49^	Preliminary; further studies needed
Lipodystrophy syndromes (with cutaneous stigmata: acanthosis nigricans, eruptive xanthomas, impaired wound healing, dermatomyositis-associated forms)	Dual GIP/GLP-1 agonism improves insulin sensitivity, reduces ectopic lipid accumulation, lowers triglycerides, suppresses glucagon	Observational cohort (n = 17): ↓BMI (-1.7 kg/m^2^), ↓HbA1c (−1.1%), ↓TG (-65 mg/dL), ↓insulin use (-109 U/day); only mild GI effects^52^	Particularly relevant when metreleptin efficacy is limited; tirzepatide may alleviate secondary skin sequelae

## Conclusion and future perspectives

Tirzepatide is an effective therapeutic option for type 2 diabetes mellitus and obesity, used either as a first-line or add-on agent depending on patient characteristics in alignment with ADA and SBD guidelines. Cutaneous side effects, primarily injection site reactions, are common but usually mild and manageable, whereas hypersensitivity and severe allergic reactions are rare but require prompt recognition and intervention. Proper patient education, injection technique, and clinical monitoring are essential to mitigate risks and optimize therapeutic outcomes. Beyond its adverse effects, tirzepatide may also offer preliminary dermatologic benefits, including potential improvements in inflammatory skin diseases such as psoriasis, hidradenitis suppurativa, as well as androgenic alopecia. These observations remain anecdotal, and controlled dermatology-focused clinical studies and registries are needed to establish efficacy.

As tirzepatide use expands, real-world data will better define its safety profile, including cutaneous effects. Future research should focus on studies to understand immune reactions at the molecular levels, identifying patient risk factors for hypersensitivity, developing formulations with reduced immunogenicity, and exploring desensitization protocols for patients who benefit metabolically but have mild hypersensitivity.

## Research data availability

The entire dataset supporting the results of this study was published in this article.

## Financial support

None declared.

## Authors' contributions

Heba Saed El-Amawy: Conceptualized the study, conducted the literature search and data analysis, wrote the original draft, reviewed and edited the manuscript, and approved the final version for submission.

## Conflicts of interest

None declared.
